# Crushing analysis of hierarchical multicellular tubes with gradient character along axial and radial directions under oblique loads

**DOI:** 10.1016/j.heliyon.2023.e13595

**Published:** 2023-02-09

**Authors:** Xiaolin Deng, Qi Lu, Fuyun Liu, Mei Liang, Qiuyun Wei, Jiale Huang

**Affiliations:** a School of Electronics and Information Engineering, Wuzhou University, Wuzhou, China; b School of Mechanical and Electrical Engineering, Guilin University of Electronic Technology, Guilin, China; c College of Foreign Languages, Wuzhou University, Wuzhou, China; d School of Mechanical Engineering, Dongguan University of Technology, Dongguan, China; e School of Mechanical and Electrical Engineering, Guangzhou University, Guangzhou, China

**Keywords:** Hierarchical structure, Multicellular column, Gradient structure, Oblique loads

## Abstract

To overcome the disadvantage of high initial peak crush force (IPCF) in hierarchical and gradient structures, the hierarchical multicellular tubes (HMTs) with gradient character along axial and radial directions are proposed based on the bidirectional structural characters of bamboo stem. Crashworthiness performances of HMTs under oblique loads are systematically studied by using numerical simulation. Results show that compared with the square tube with the same mass, HMTs have higher energy absorption capability under different impact angles. The maximum increases of specific energy absorption (SEA) and crush force efficiency (CFE) reach up to 67.02% and 806%, respectively. Whereas, the maximum decrease of IPCF reaches up to 79.92%. Effects of structural parameters, including hierarchical level, wall thickness and internode space, on the crashworthiness performances of HMTs are also fully investigated.

## 1 Introduction

The huge number of cars leads to the frequent occurrence of traffic accidents, which not only causes significant economic losses, but also causes serious physical and mental injuries to the passengers. Therefore, improving the safety as much as possible during collision is the important goal in the vehicle design process. The important way for enhancing the passive safety of the vehicles is to improve the energy absorption capacity and efficiency. Energy absorption structures should have higher energy absorption efficient and regular deformation patterns as well as low peak crush force (PCF). Thin-walled metal tubes are widely used in the energy absorption structures of automobiles, aircraft and high-speed railway vehicle. When a collision occurs, thin-wall tubes will appear plastic deformation, extreme interactions and material fracture, which absorbs mass of kinetic energy [1,2].

The common used thin-walled structures, including circular tubes [3], square tubes [4], star-shaped tubes [5] and multi-cell hexagonal tubes [6], have been extensively investigated. Hierarchical structures are the novel absorption structures, which has gained attention recently. Several studies of hierarchical structures via numerical simulation [7,8], experiment [[9], [10], [11]], theoretical analysis [12] and optimization method [13] have been carried out. By substituting the vertex of hexagon honeycomb into small hexagon honeycomb, Du and Hao et al. [14] designed a three hierarchical level structure and numerically investigated its crashworthiness performances. Results show that the proposed hierarchical honeycomb has high energy absorption capacity. Based on this study, they used small hexagon honeycomb to replace the middle of cell rib and both of the vertex of three cell rib and middle of cell rib original honeycomb [15] to form different types of hierarchical honeycombs. Numerical results show that the energy absorption capacity of hierarchical honeycomb with replacing both the vertex and middle of cell ribs into small honeycomb is the best, and the performance of hierarchical honeycomb generated by substituting the middle cell ribs is better than that by substituting the vertex. Li et al. [16] designed and numerical investigated the hierarchical cellular structures with negative Poisson's ratio. Results show that the second order of hierarchical structure significantly increases the energy absorption due to the prevention of local deformation during impact process. Rahman et al. [17] found that although the hierarchical characterizes increase the in-plane stiffness, the elastic modulus of structure will be more sensitive to the defects. Wang et al. [8] proposed the vertex-based hierarchical square thin-walled multi-cell structure and derived its mean crush force via classic angle-fold element theory. Combining the auxetic structure and hierarchical property, Tan et al. [18] designed two types of re-entrant hierarchical honeycombs with negative Poisson's ratio by replacing the cell walls of re-entrant honeycombs with regular hexagon substructure (RHH) and equilateral triangle substructure (RHT). Numerical results show that the specific energy absorption of RHT and RHH is improved by up to 292% and 105%. The aforementioned studies clearly showed the superiority of hierarchical structure in energy absorption.

Recently, various gradient structures have been widely studied and developed in order to control the initial peak force during the impact process. Biomechanical studies show that the tolerance of human head can be expressed by Eq. (1) [19].

(1) $$GSI=\int_{0}^{T}a^{2.5}dt<1000$$

GSI is the *Gadd Severity Index*, which is related to the acceleration (or deceleration) *a*. Thin-walled structure reaches the initial peak force in a very short time at the beginning of collision. Therefore, to decrease its acceleration *a*, the initial peak force must be maintained at a low value. In order to reduce the initial peak force of the structure, scholars proposed structures, including conical tube with various structural parameters [[20], [21], [22]], gradient variable thickness tube along radial [23] and axial [24,25] directions. Another important way to realize the gradient structure is through changing the material properties, such as gradient density and gradient strength [26,27].

Based on the hierarchical structure of bamboo, Ha et al. [28] proposed a bio-inspired hierarchical multi-cell square tubes, investigated its crashworthiness performances under axial impact via numerical simulation and calculated the mean crush force by using simplified super folding element theory. Results show that the specific energy absorption (SEA) of bio-inspired hierarchical multi-cell square tubes increases by 173.7% and 128.1% compared with the traditional square tube and multi-cell square tube. Based on the spatial folding principle, a novel gradient hierarchical multicellular column was designed by folding the smallest and outermost region of the cross section to generate the next level of structure [29]. Numerical results show that the SEA and crush force efficiency (CFE) increase by 72.44% and 97.66%, respectively, compared with the square tube under the same mass.

The aforementioned studies clearly show that both the hierarchical structures and the gradient hierarchical structures (along the radial direction) have great advantage in energy absorption compared with the traditional thin-walled structures. However, these structures are not benefit in reducing the initial peak crush force (IPCF). Excessive IPCF causes great damage to passengers or cargos during crash. Nevertheless, the existing structure mainly reduces its IPCF through the methods of changing the diameter/width and thickness of structure, density and strength of materials and introducing initial imperfection. Besides, the crashworthiness studies of thin-walled tubes with gradient structural design along the axial direction are rarely reported. To further decrease the IPCF of gradient hierarchical structures and maintain their high energy absorption character, the hierarchical multicellular tubes with gradient character along axial and radial directions are proposed in this study. To the best of our knowledge there are not similar literatures designing the bidirectional gradient hierarchical thin-walled structure. The crashworthiness performances of this structure under oblique loading have been fully investigated. The proposed thin-walled structure can be used in the fields of energy absorption devices, such as automotive, aerospace and civil engineering. These results can serve as guides for the crashworthiness design of bidirectional gradient hierarchical thin-walled structures.

## 2 Materials and methods

### 2.1 Structural design

**Fig. 1:**
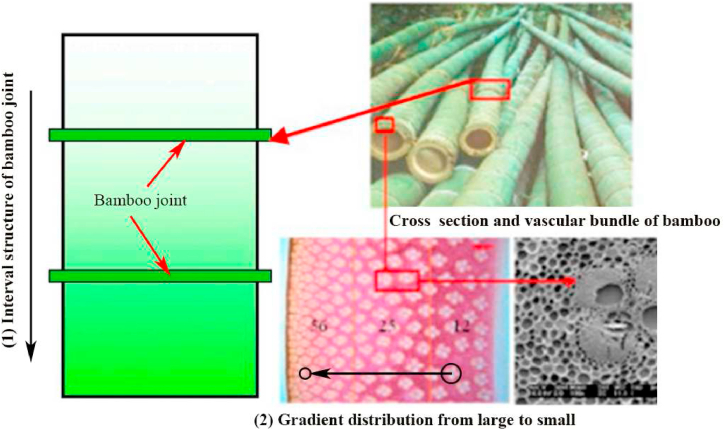
The gradient and hierarchical structures of bamboo [31].

Bamboo is a worldwide-used natural engineering material, which is famous for its high strength and excellent toughness. The micro materials distribution presents gradient and hierarchal characteristics along the radial and axial directions. Such multi-level hierarchical composited structures are the key for the excellent performances of bamboo [30]. Inspired by the bidirectional gradient structure of bamboo, the hierarchical multicellular tubes (HMT) with gradient character along radial and axial directions (Fig. 1) are proposed in this study to decrease the PCF of the hierarchical structures while maintaining its high energy absorption performance.

**Fig. 2:**
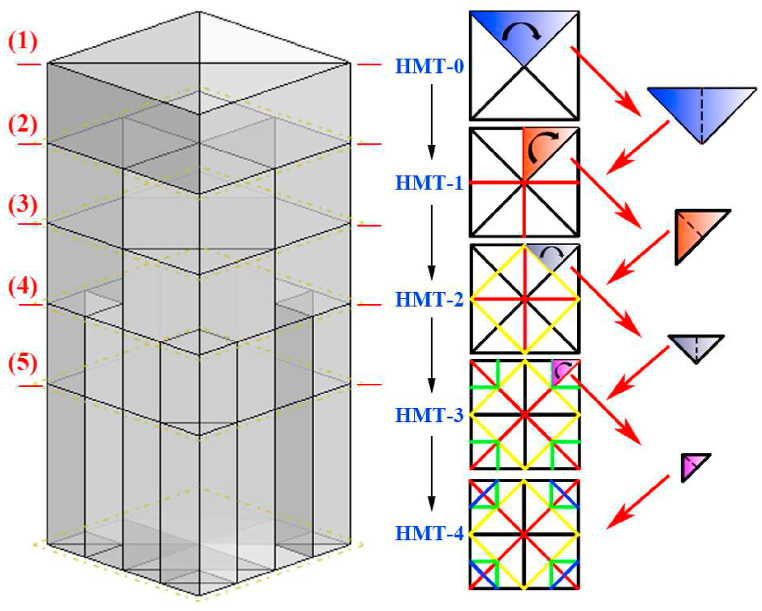
Design principles of hierarchical multicellular tubes.

**Fig. 3:**
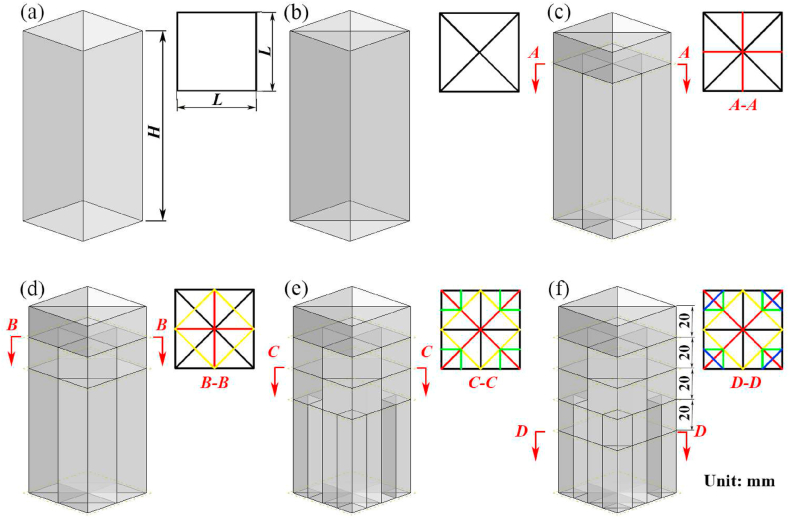
Geometry design of HMTs, (a) square tube, (b) HMT-0, (c) HMT-1, (d) HMT-2, (e) HMT-3 and (f) HMT-4.

The evolvement of the proposed HMT with bidirectional gradient materials distribution is shown in Fig. 2. The HMTs with different hierarchical levels are named as “HMT-n” and their 3- dimensional views are shown in Fig. 3 (a, b, c, d, e, f). The height and side length of the HMTs are fixed as 120 mm and 50 mm, respectively. HMT-0 is composed of a square tube and a crisscross rib plate (Fig. 3b). Based on the structure of HMT-0, the element of triangle region is folded to generate the cross section of next level of hierarchical structure (HMT-1). Within the distance of 20 mm from the tip, the cross section of the hierarchical structure is HMT-0, and below the distance of 20 mm, the cross section changes to HMT-1, which forms a gradient structural distribution along the axial direction, as shown in Fig. 3c. The smallest and outermost region of the cross section is folded to generate the next level of the novel hierarchical multicellular structure and they are sequentially placed on the axial position (20 mm intervals) to construct the bidirectional gradient hierarchical tube, as shown in Fig. 3. Such gradient distribution of materials along the axial direction decreases the material mass near the impact end, which is expected to reduce the peak crush force at the initial stage of impact process. Meanwhile, the multilayer hierarchical structure along the radial direction maintains the excellent energy absorption capability. Therefore, the proposed bidirectional gradient hierarchical multicellular tube is expected to serve as a candidate choice for the design of high performance energy absorber.

### 2.2 Finite element simulation

#### 2.2.1 Construction of finite element model

**Fig. 4:**
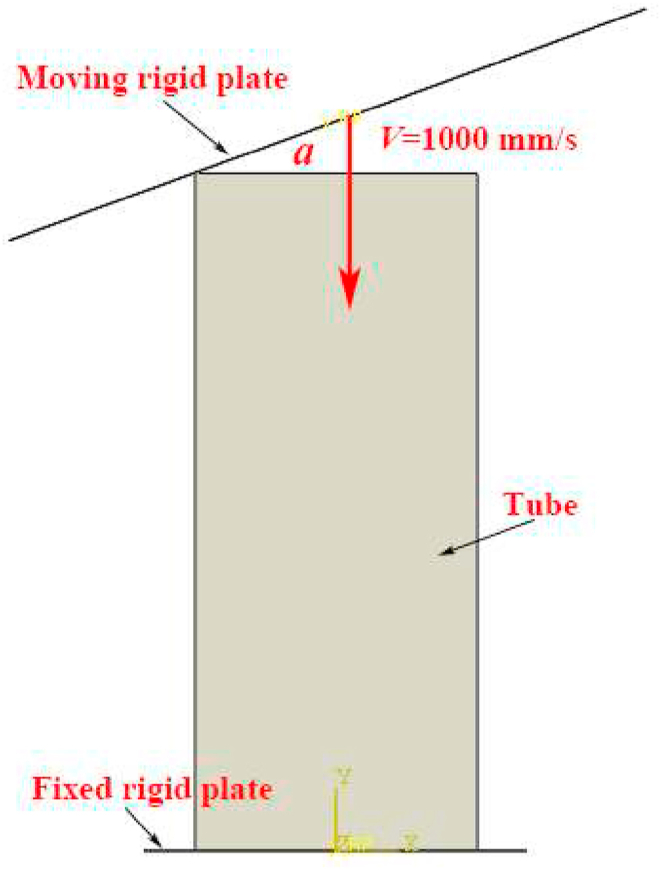
Schematic of impact model.

Finite element models are constructed by using Abaqus/Explicit software and the schematic diagram of impact process is shown in Fig. 4. The models are composed of three parts, including moving rigid plate, hierarchical multicellular tube and fixed rigid plate. The moving rigid plate smacks toward the thin-walled tube with a preset angle α. The mass of moving rigid plate is set as 600 kg and the impact velocity is fixed as 1000 mm/s. The impact distance is set as 85% of the total height of hierarchical multicellular tubes *H* (120 mm × 85% = 102 mm). The moving rigid plate and fixed rigid plate are set as analytic rigid body. Four-node reduced integral shell element S4R was used to simulate the HMTs and 5 integration points are used along the thickness direction. The HMTs were boned to the fixed rigid plate, and all degrees of freedom of the fixed plate are constrained. General contact was applied to the model to describe the interaction effect and the friction coefficient is set as 0.2. The material properties of aluminum alloy AA6063-T5 are the same of reference [28], with the density ρ = 2700 kg/m^3^, elastic modulus *E* = 70 GPa, Poisson's ratio *μ* = 0.3, yield stress of 219 MPa and the ultimate stress of 250 MPa. Considering that the AA6063-T5 is low sensitive to the strain rate, the effect of strain rate on the materials is neglected in this study.

#### 2.2.2 Crashworthiness index

**Fig. 5:**
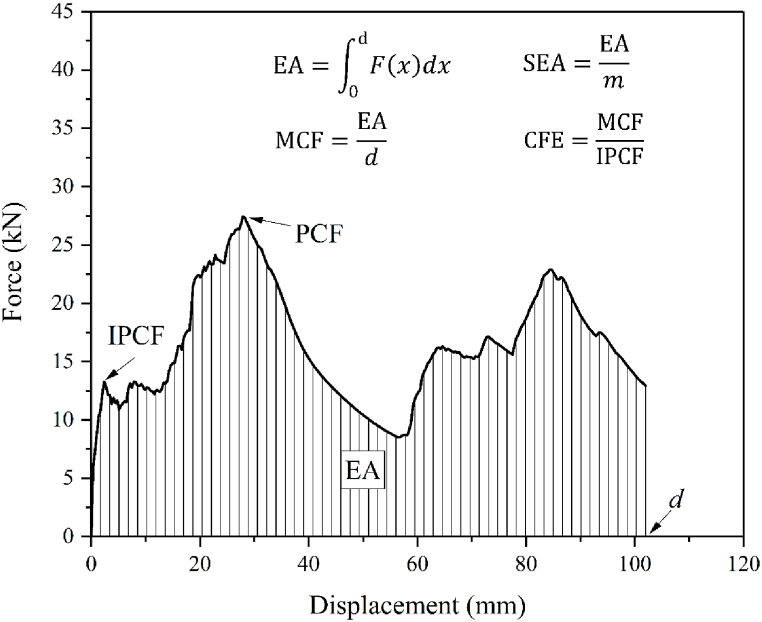
Indexes of crashworthiness performances.

To quantificationally evaluate the energy absorption performances of structure, six indexes, including energy absorption (EA), specific energy absorption (SEA), mean crush force (MCF), initial peak crush force (IPCF), peak crush force (PCF) and crush force efficiency (CFE), were used in this study, as shown in Fig. 5. They can be calculated by Eqs. (2), (3), (4), (5).

(2) $$\text{EA}=\int_{0}^{d}F\left(x\right)dx$$

(3) $$SEA=\frac{EA}{m}$$

(4) $$MCF=\frac{EA}{d}$$

(5) $$CFE=\frac{MCF}{IPCF}\times 100\%$$

where, *F*(*x*) is the instantaneous impact force, *d* is the impact distance and *m* is the structure mass. For the axial impact process, the PCF generally occurs at the initial stage of collision, and thus, it is the same as the IPCF. Whereas, for the oblique impact, the peak crush force generally appears at the latter stage of the impact process (at this situation, PCF is larger than IPCF). It must be pointed out that although PCF is larger than IPCF, the acceleration is extremely large, leading to the high GSI according to Eq. (1). Therefore, the damage for human body during collision is dominated by IPCF. The CFE is determined by the ratio of MCF to IPCF in this paper. If CFE is larger than 1, the MCF is larger than the IPCF. Excellent energy absorber should have high EA, SEA, MCF, CFE and low IPCF.

#### 2.2.3 Convergence analysis

**Fig. 6:**
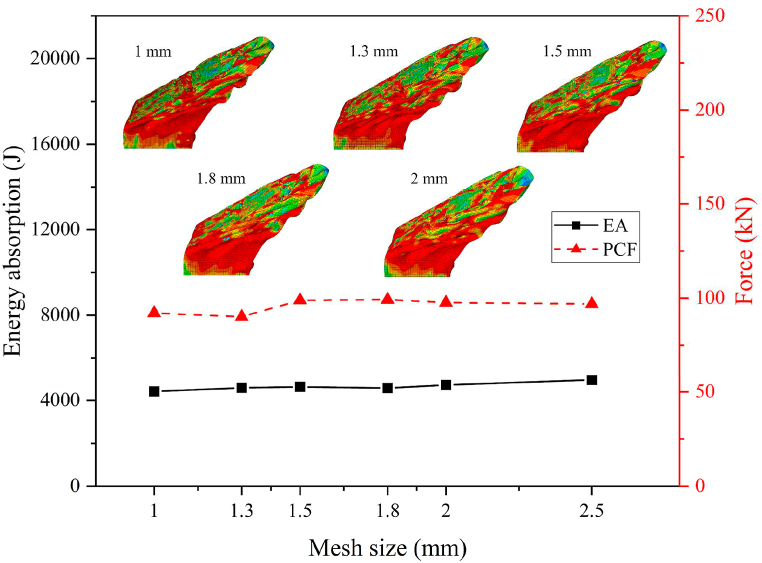
Convergence analysis of finite element model.

Convergence analyses were carried out by using the HMT-4 with 1 mm thickness under the 30° oblique impact loading. The mesh sizes of 1 mm, 1.3 mm, 1.5 mm, 1.8 mm, 2 mm and 2.5 mm were adopted. The results are shown in Fig. 6 and Table 1. The plot shows that the mesh size has great influence on the numerical results. However, when the mesh size is 1.3 mm, the difference of energy absorption is only 4.46% compared with the mesh size of 1 mm, and the difference of PCF is only −2.14%. Moreover, the deformation modes of HMT-4s with different mesh sizes show a very high level of similarity. Note that the structure of HMT-4 is the most complex, meanwhile, the deviations of energy absorption and PCF between HMT-4s with mesh sizes of 1.3 mm and 1 mm are both less than 5%. Therefore, considering the computing time and the accuracy of the numerical analysis, the mesh size of 1.3 mm was adopted in this study.

**Table 1:** Detailed data of the convergence analysis.

Mesh size (mm)	EA (J)	Diff (%)	PCF (kN)	Diff (%)
1.0	4418.89	–	92.11	–
1.3	4595.83	4.46	90.13	−2.14
1.5	4638.89	10.99	98.87	7.35
1.8	4580.49	17.99	99.23	7.73
2.0	4722.98	23.81	97.69	6.07
2.5	4966.81	32.69	96.89	5.19

#### 2.2.4 Validation of finite element model

The accuracy of finite element model was validated by the simplified super folding element (SSFE) theory. According to the energy balance of the system, Eq. (6) can be obtained.

(6) $$2∙MCF∙H∙k=W_{bending}+W_{membrane}$$

where, *MCF* is the mean crush force, *H* is the half of wavelength, *k* is the coefficient of effective crushing distance and it is set as 0.7 in the study [32]. *W*_bending_ and *W*_membrane_ are the bending energy and membrane energy, respectively.

The bending energy *W*_bending_ is calculated by the sum of three plastic hinge lings, as shown in Eq. (7).

(7) $$W_{bending}=\sum_{i=1}^{3}{\theta_{i}M}_{0}L_{c}$$

where $M_{0}=\frac{1}{4}\sigma_{0}t^{2}$ is the fully plastic bending moment, *σ*_0_ is the flow stress which can be obtained by $\sigma_{0}=\sqrt{\frac{\sigma_{y}\sigma_{u}}{1+n}}$. *σ*_y_ is the yield stress, *σ*_u_ is the ultimate stress, *n* is the strain hardening exponent and it is set as 0.06 in this study.

The total bending energy can be calculated by Eq. (8).

(8) $$W_{bending}=2\pi M_{0}L_{c}$$

*L*_c_ is the perimeter of cross section and *t* is the wall thickness.

**Fig. 7:**
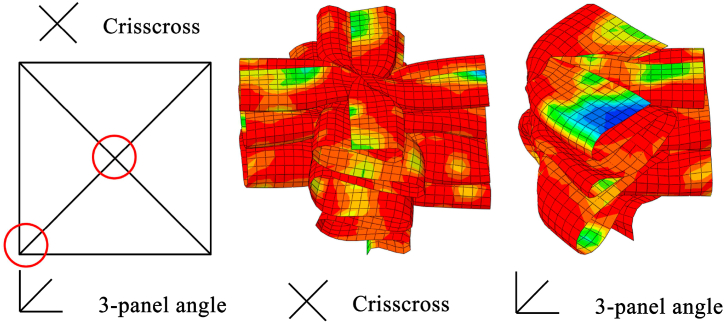
The cross sectional shape and basic elements of HMT-0.

The membrane energy dissipations of different basic elements are also different. Here, the MCF of HMT-0 is used to validate the accuracy of finite element model. The corresponding cross sectional shape and basic element of HMT-0 are shown in Fig. 7.

The membrane energy of 3-panel element can be calculated by the sum of corner element and an addition 1-panel element [33], as shown in Eq. (9).

(9) $$E_{3-panel}=4M_{0}\frac{H^{2}}{t}\left(\frac{\tan\;\alpha }{\left(\tan\;\alpha +0.05/\tan\;\alpha \right)/1.1}+2\;\tan\frac{\alpha }{2}\right)$$

The membrane energy of crisscross element, which is composed of two symmetric right angle elements, can be described by Eq. (10) [34].

(10) $$E_{crisscross}=2E_{right\;angle}=16M_{0}\frac{H^{2}}{t}$$

The membrane energy of GHMC-0 can be sum of each basic element, as shown in Eq. (11).

(11) $$W_{membrane}=n_{3}E_{3-panel\;angle}+n_{C}E_{crisscross}$$

where *n*_3_ and *n*_c_ are the number of 3-panel angle element and crisscross element. For the GHMC-0, the numbers of 3-panel angle element and crisscross element are 4 and 1, respectively. Thus, the total membrane energy is obtained as follows.

(12) $$W_{membrane}^{sum}={7.876n}_{3}M_{0}\frac{H^{2}}{t}+{16n}_{C}M_{0}\frac{H^{2}}{t}=\left({7.876n}_{3}+{16n}_{C}\right)M_{0}\frac{H^{2}}{t}$$

Combining Eqs. (6), (8), (12), Eqs. (13), (14) can be obtained.

(13) $$2HkMCF=2\pi M_{0}L_{c}+\left({7.876n}_{3}+{16n}_{C}\right)M_{0}\frac{H^{2}}{t}$$

(14) $$MCF=\frac{1}{k}\left(\frac{\pi M_{0}L_{c}}{H}+\left({3.752n}_{3}+{8n}_{C}\right)M_{0}\frac{H}{t}\right)$$

According to the stationary condition $\frac{\partial MCF}{\partial H}=0$, the half of wavelength *H* can be calculated by Eq. (15).

(15) $$H=\sqrt{\frac{\pi L_{c}t}{\left({3.752n}_{3}+{8n}_{C}\right)}}$$

Substituting Eq. (15) into Eq. (14), the expression of *MCF* can be obtained by Eqs. (16), (17).

(16) $$MCF=\frac{2}{k}M_{0}\sqrt{\frac{{\left({3.752n}_{3}+{8n}_{C}\right)\pi L}_{c}}{t}}$$

(17) $$MCF=\frac{1}{2k}\sigma_{0}t\sqrt{{\left({3.752n}_{3}+{8n}_{C}\right)\pi L}_{c}t}$$

Because the impact velocity is set as 1000 mm/s, the dynamic enhancing coefficient $\lambda_{i}$ = 1.1 is introduced to consider the inertia effect [28]. Therefore, the expression of modified MCF is calculated by Eq. (18).

(18) $$MCF=\frac{\lambda_{i}}{2k}\sigma_{0}t\sqrt{{\left({3.752n}_{3}+{8n}_{C}\right)\pi L}_{c}t}$$

**Fig. 8:**
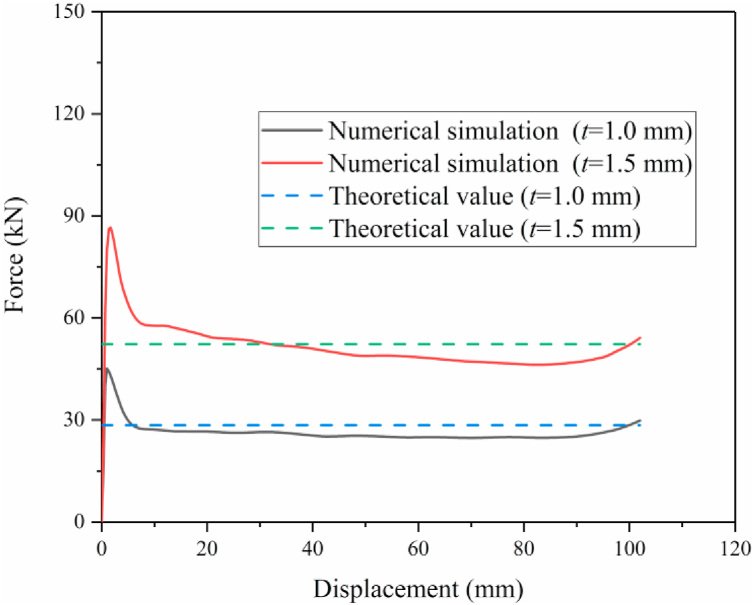
Validation of finite element model.

Fig. 8 shows the relationship between MCF and displacement of HMT-0 with the wall thickness of 1 mm and 1.5 mm. The plot shows that when the impact distance is 102 mm, the numerical and theoretical results of MCF are 29.89 kN and 28.47 kN, which difference is only 4.99%. For the HMT-0 with 1.5 mm wall thickness, the numerical and theoretical results of MCF are 54.17 kN and 52.30 kN, which difference is only 3.58%. Therefore, the proposed finite element model has enough accuracy, which can be used in the subsequent numerical analysis.

## 3 Comparative analysis of crashworthiness performance

**Fig. 9:**
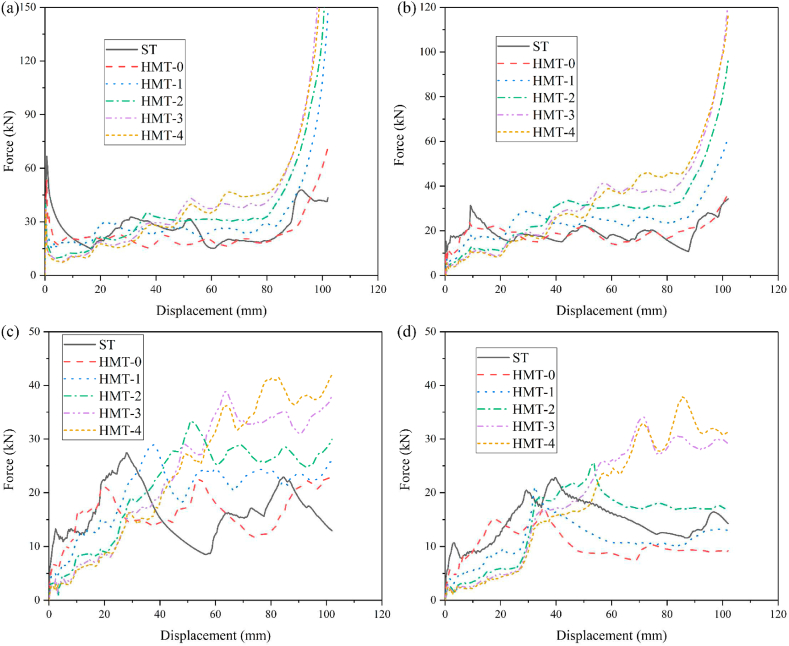
Force-displacement of HMTs, (a) 0°, (b) 10°, (c) 20° and (d) 30°.

In order to compare the energy absorption of HMT with common square tube, the thickness of square tube is set as 1.5 mm and the wall thickness of HMTs with different hierarchical levels are changed to maintain the same mass as the square tube. The crashworthiness performances of HMTs and square tube under different impact angles are shown in Fig. 9 (a, b, c, d) and Table 2. When the impact angle is equal to 0°, the impact force of square tube reaches peak value rapidly at the initial stage and then drops down and enters to the plateau region, which is similar to other conventional hollow thin-walled tubes. However, large value of PCF is unfavorable for the protection of passenger during collision. Unlike the conventional square tube, as the hierarchical level increases, the IPCF continuously decreases. For the structure of HMT-4, the IPCF is only 36.86 kN under the impact angle of 0°, which decreases by 44.67% compared with that of square tube (66.62 kN). In addition, the impact force of HMT shows a gradual increasing trend during the impact. From the point of view of automobile engineering, these characters are beneficial to the energy absorption, because the low IPCF decreases the acceleration at the initial stage, avoiding the harm for the passenger, and the impact force increases gradually at the latter stage of impact process, which enhances the total energy absorption of the structure.

**Table 2:** Crashworthiness data of HMTs with the same mass.

No	*a* （°）	*t* (mm)	*m* (kg)	EA (J)	SEA (kJ/kg)	IPCF (kN)	CFE (%)
ST	0	1.5	0.0972	2671.78	27.49	66.62	39.32
ST	10	1.5	0.0972	1937.11	19.93	15.31	124.02
ST	20	1.5	0.0972	1529.18	15.73	13.27	112.98
ST	30	1.5	0.0972	1249.29	12.85	10.67	114.80
HMT-0	0	0.8787	0.0972	2286.17	23.52	53.33	42.03
HMT-0	10	0.8787	0.0972	1838.22	18.91	10.54	171.00
HMT-0	20	0.8787	0.0972	1515.27	15.59	6.74	220.28
HMT-0	30	0.8787	0.0972	833.10	8.57	5.65	144.57
HMT-1	0	0.7063	0.0972	3093.96	31.83	40.84	74.27
HMT-1	10	0.7063	0.0972	2426.37	24.96	7.78	305.79
HMT-1	20	0.7063	0.0972	1859.74	19.13	4.61	395.87
HMT-1	30	0.7063	0.0972	909.31	9.36	3.98	223.75
HMT-2	0	0.5780	0.0972	3500.95	36.02	39.83	86.18
HMT-2	10	0.5780	0.0972	2856.40	29.39	4.02	696.12
HMT-2	20	0.5780	0.0972	1938.30	19.94	3.64	522.32
HMT-2	30	0.5780	0.0972	1221.54	12.57	2.47	484.57
HMT-3	0	0.5272	0.0972	4099.14	42.17	37.71	106.57
HMT-3	10	0.5272	0.0972	3211.13	33.04	5.62	560.60
HMT-3	20	0.5272	0.0972	2191.12	22.54	2.67	806.00
HMT-3	30	0.5272	0.0972	1644.58	16.92	2.85	566.07
HMT-4	0	0.5062	0.0972	3901.96	40.14	36.86	103.77
HMT-4	10	0.5062	0.0972	3235.39	33.29	5.72	554.39
HMT-4	20	0.5062	0.0972	2191.99	22.55	2.75	780.74
HMT-4	30	0.5062	0.0972	1632.25	16.79	2.44	656.36

**Fig. 10:**
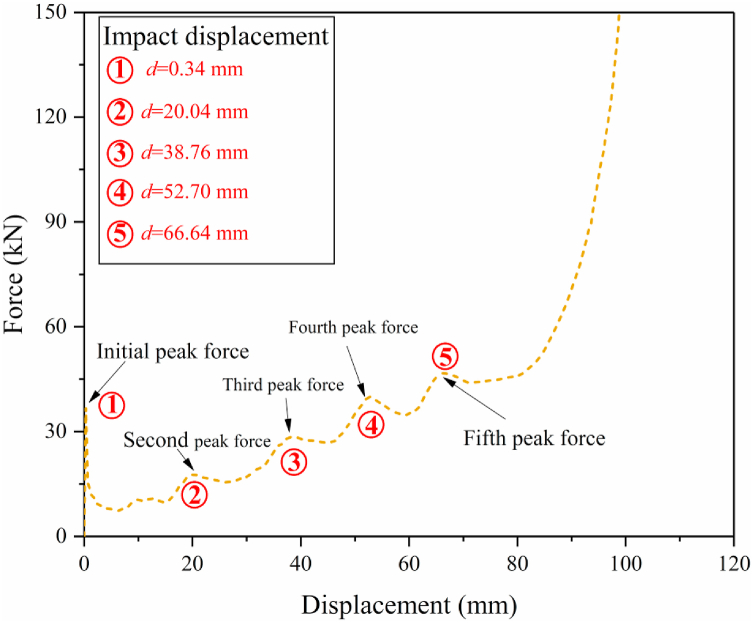
Force-displacement of HMT-4.

**Fig. 11:**
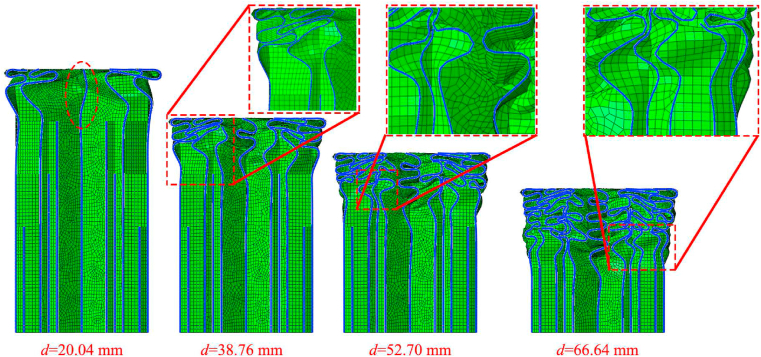
Deformation patterns of HMT-T at the critical peak force positions.

To better analyze the change of impact force of HMT, the force-displacement curve of HMT-4 under the impact angle of 0° is shown in Fig. 10, and the structural deformations at the key positions are indicated. The plot shows that the impact force immediately reaches maximum value at the initial stage and then decreases rapidly. When the impact distance reaches 20.04 mm, the second peak force occurs. This peak force in the curve is attributed to the additional rib of the 2nd level at the distance of 20 mm from the tip (Fig. 3), which increases the number of ribs with plastic deformation, leading a suddenly change of force at this moment. Similarly, three peak forces consecutively occur at the displacement of 38.76 mm, 52.70 mm ad 66.64 mm. Therefore, the impact force can continuously increase through laying up materials with gradient distribution along axial direction. It must be pointed out that as the tube continuously to be compressed, materials gradually collapse from top to down and the new ribs will be involved in the plastic deformation process in advance. Therefore, these three peak values do not precisely occur at the positions of 40 mm, 60 mm and 80 mm. Taking the d = 52.70 mm as example (Fig. 11), although the impact distance does not reach 60 mm, the additional rids at the position of 60 mm has been bent, suggesting that they have been involved in the plastic deformation in advance.

**Fig. 12:**
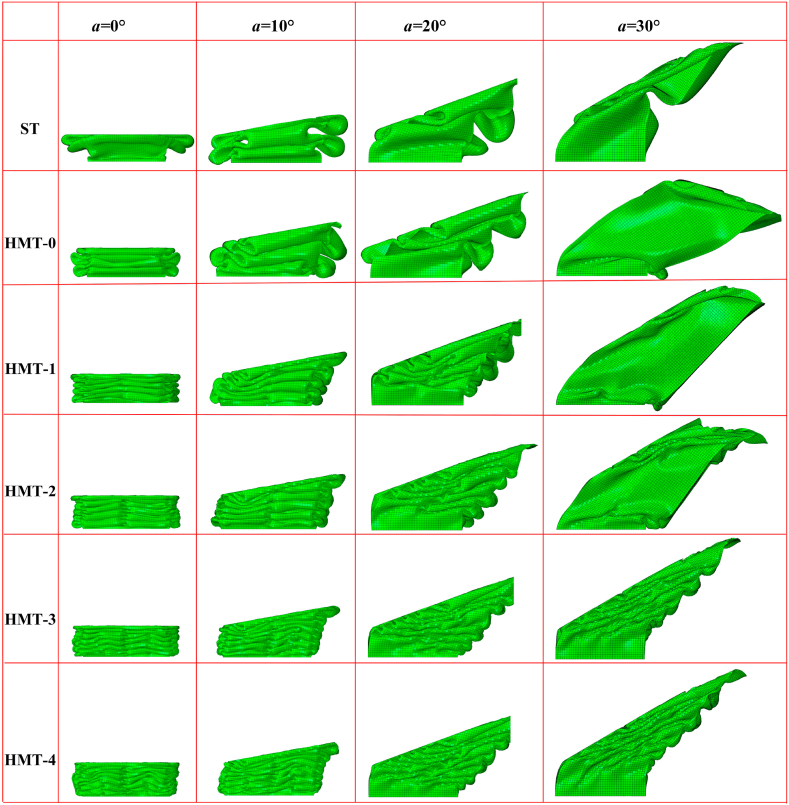
Final deformation patterns of HMTs under impact at different angles.

**Fig. 13:**
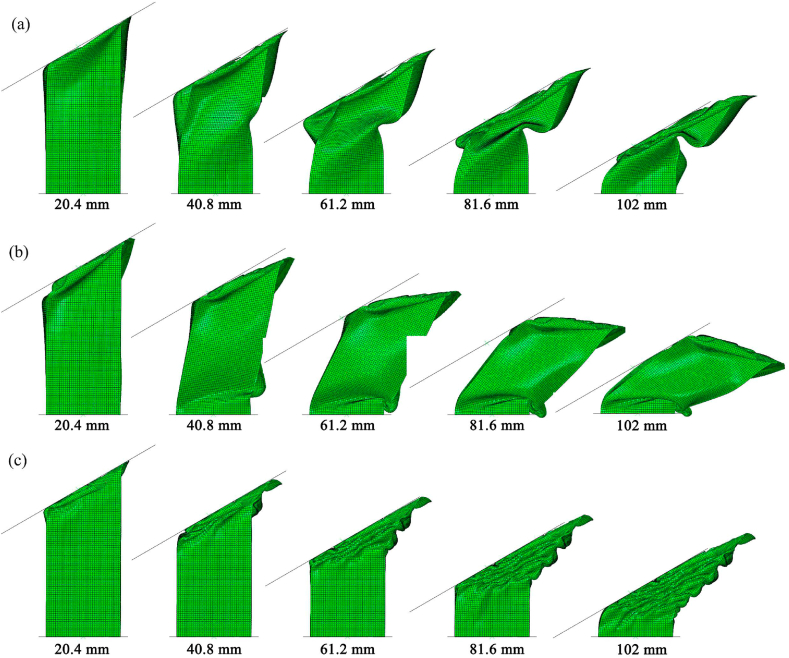
Deformation process of final deformation of three typical structures (a) square tube, (b) HMT-0 and (c) HMT-4.

**Fig. 14:**
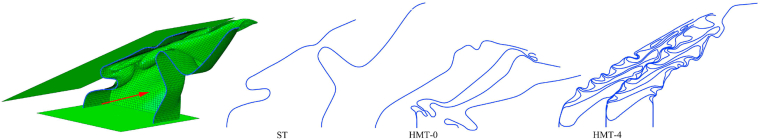
Cross section shape of final deformation of three typical structures.

**Fig. 15:**
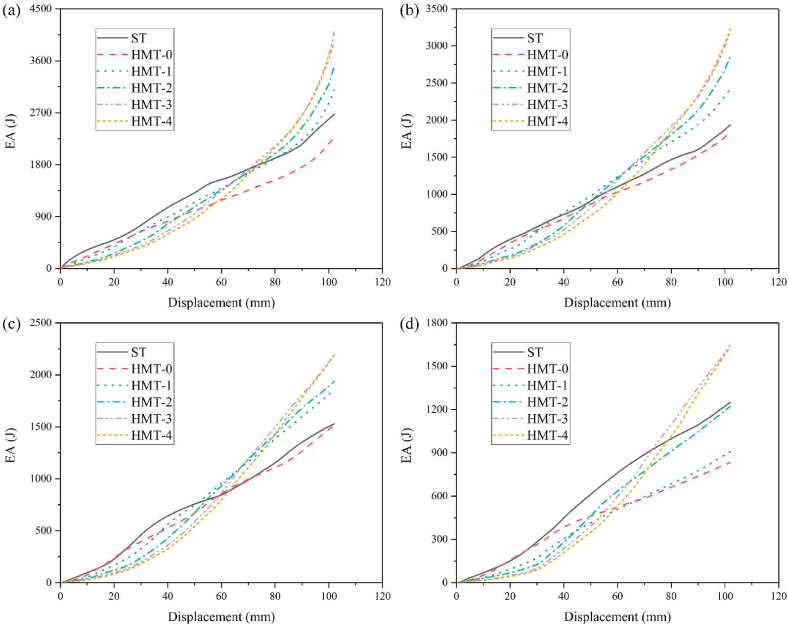
Energy absorption of HMTs under different impact angles (a) 0°, (b) 10°, (c) 20° and (d) 30°.

The IPCF of HMTs not only will be significantly decreased under the axial impact but also have substantial advantages under the oblique impact. The final deformation patterns of different HMTs under different impact angles are shown in Fig. 12. The deformation process and the cross section shape of final deformation of three typical structures (ST, HMT-0 and HMT-4) are shown in Fig. 13 (a, b, c) and Fig. 14. Fig. 12 shows that the number of folds is the most under the impact angle of 0°, and all structures produce steady progressive folding mode. When the impact angle increases to 10°, although all structures still produce progressive folding mode, the numbers of folds of square tube and HMT-0 are less than those of HMT-3 and HMT-4. The HMT-4 produces 7 complete folds while the square tube only produces 2 folds under the impact angle of 10°. As the impact angle increases, the deformation mode difference among these structures increases. When the impact angle is equal to 30°, HMT-0, HMT-1 and HMT-2 generate overall bending mode, which has low energy absorption performance. Whereas, HMT-3 and HMT-4 still generate the progressive folding mode. Moreover, unlike the large folds of square tube, the folds produced by the HMT-3 and HMT-4 are not only numerous but also have small wavelength. The energy absorption curve in Fig. 15d also shows that the energy absorptions of HMT-0, HMT-1 and HMT-2 are relatively low while HMT-3 and HMT-4 have higher energy absorption performance. This phenomenon is mainly attributed to the thinner wall thickness of HMT (especially for the high hierarchical level), leading to the larger number of folds and higher energy absorption. Table 2 shows that compared to the square tube of 1.5 mm, the wall thickness of HMT-4 decreases to 0.5062 mm, therefore, the end of HMT-4 near the impact plane is easier to produce progressive folding mode. Fig. 15 (a, b, c, d) also shows that at the early stage, the energy absorption of HMTs is generally not as good as that of square tube, however, as the impact distance increases, the energy absorption of HMTs increases rapidly. When the impact distance reaches 102 mm, HMTs have the best performance regardless the angle of the impact. These characters clearly show that HMTs not only have absolute advantages in reducing IPCF, but also in increasing the energy absorption compared with square tube.

**Fig. 16:**
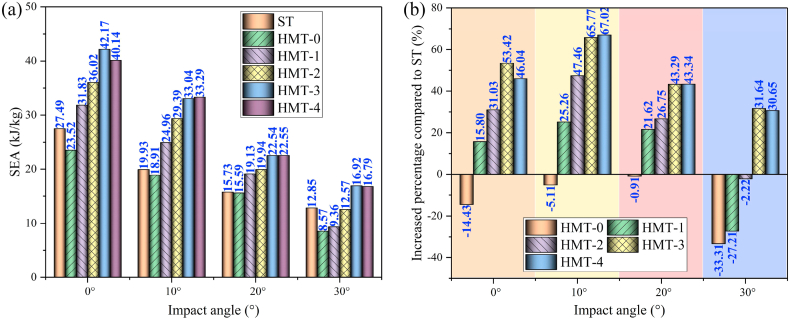
SEA analysis of different structures (a) SEA and (b) increased percentage compared with square tube.

**Fig. 17:**
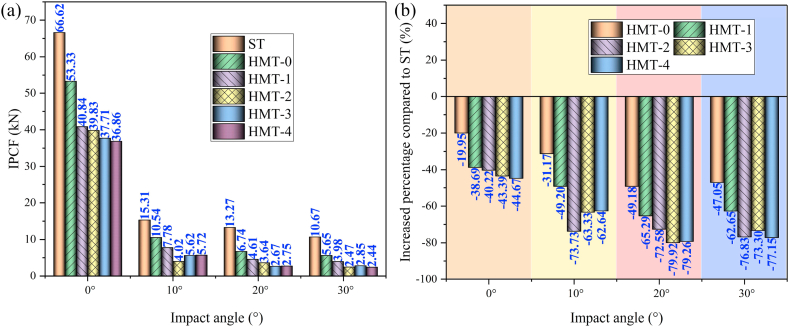
IPCF analysis of different structures (a) IPCF and (b) increased percentage compared with square tube.

**Fig. 18:**
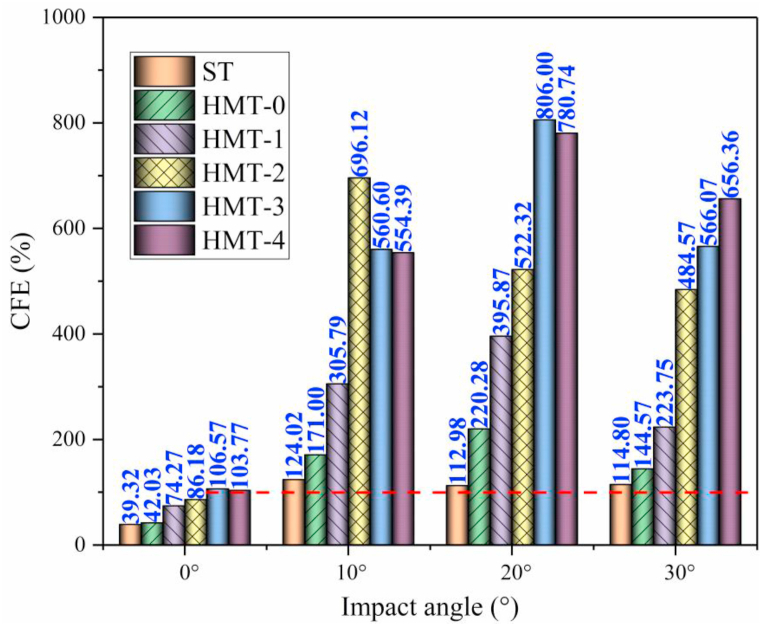
CFE analysis of different structures.

Fig. 16 (a, b), Fig. 17 (a, b) and Fig. 18 show the crashworthiness indexes of HMTs and square tube. Obviously, HMTs have higher energy absorption compared with square tube. Especially for the HMT-3 and HMT-4, the SEA of HMT-3 and HMT-4 is higher than square tube under all impact angles. When the impact angle is equal to 10°, the improvement of HMT-3 and HMT-4 can reach by 65.77% and 67.02%, respectively. Moreover, HMTs have absolute advantage in IPCF, which is another key crashworthiness index. The minimum decrease of IPCF is 19.95% and the maximum decrease reaches up to 79.92% compared with square tube. Larger impact angle leads to lower IPCF of the structure. When the impact angle is equal to 30°, the IPCF of HMT-4 is only 2.44 kN, while that of square tube reaches up to 10.67 kN, which is around 4.37 times as large as that of HMT-4. Moreover, compared with the CFE of square tube (the maximum is only 114.8%), the highest CFE of HMT-3 under 20° impact angle reaches up to 806%, suggesting that the mean crash force is 8.06 times as large as the IPCF. Here, the value of red line in Fig. 18 is 100%. The plot shows that when the impact angle is equal to 10°, 20° and 30°, the value of CFE is higher than 100%, and when the impact angle is equal to 0°, only the CFEs of HMT-3 and HMT-4 exceed 100%. The aforementioned data analysis of the key crashworthiness indexes (including SEA, IPCF and CFE) clearly show that HMT has excellent crashworthiness performances. To further investigate the relationship between the crashworthiness performances and structural parameters, parametric study is carried out in the following section.

## 4 Parametric analysis

### 4.1 Effect of hierarchical level on the crashworthiness performances

**Fig. 19:**
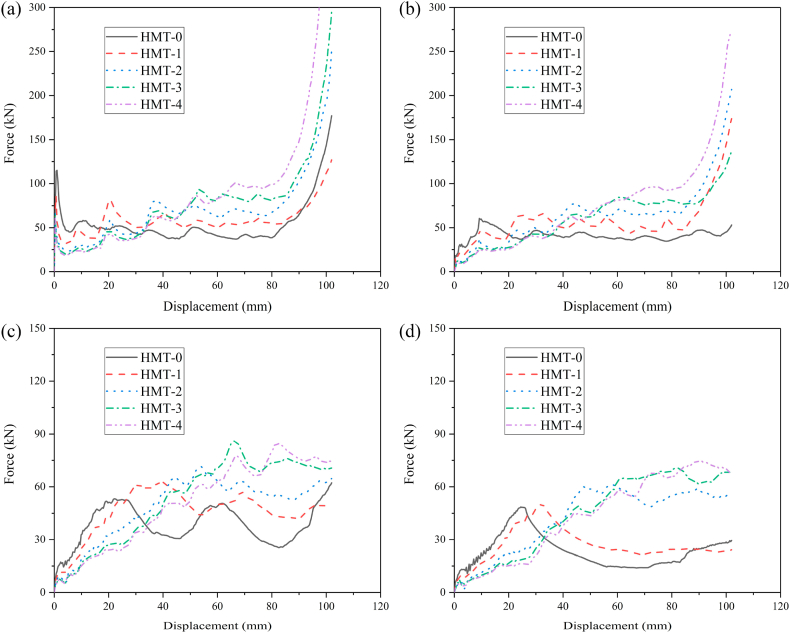
Force-displacement curves of HMTs with different hierarchical levels, (a) 0°, (b) 10°, (c) 20° and (d) 30°.

**Fig. 20:**
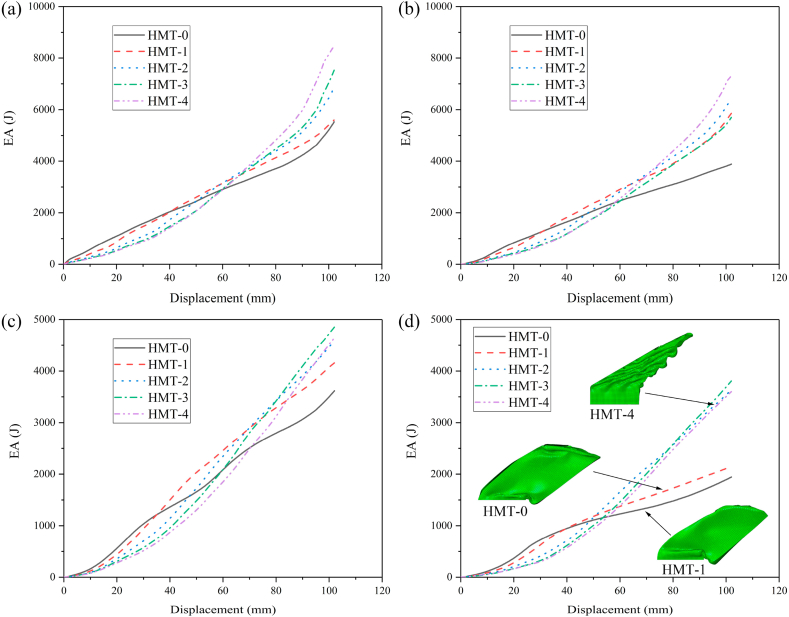
Energy absorption curves of HMTs with different hierarchical levels, (a) 0°, (b) 10°, (c) 20° and (d) 30°.

In order to analyze the effect of hierarchical level on the crashworthiness performances of HMTs, the wall thickness of HMT-0 is set as 1.5 mm and the wall thickness of other HMTs is changed to keep the same mass as the HMT-0 (0.166 kg). Detailed crashworthiness data is shown in Table 3. The force-displacement and energy absorption curves of HMTs with different hierarchical levels under different impact angles are shown in Fig. 19 (a, b, c, d) and Fig. 20 (a, b, c, d). When the impact angle is equal to 0°, the impact forces of HMTs with different hierarchical levels are smooth and steady and all of them have relative high IPCF. At the early stage of impact process, the energy absorptions of HMTs with low hierarchical levels are better than those with high hierarchical levels. However, HMTs with high hierarchical levels present better energy absorption performance at the late stage because more materials are involved in the plastic deformation. Similar to the impact angle of 0°, the energy absorption of HMT-4 is the highest under the impact angle of 10°. However, when the impact angles are equal to 20° and 30°, the impact forces of HMT-0 and HMT-1 are notable changed and the amplitudes of fluctuation are larger than those of HMTs with higher hierarchical levels. Especially under the impact angle of 30°, the impact forces of HMT-0 and HMT-1 only produce one large fluctuation, whereas, those of HMT-2, HMT-3 and HMT-4 present a gradually increased process. This phenomenon is mainly attributed to the change of deformation mode. HMT-0 and HMT-1 produce the overall bending deformation, meanwhile those of HMT-2, HMT-3 and HMT-4 are still the progressive folding mode (Fig. 20d). Correspondingly, the energy absorption of HMT-0 and HMT-1 are significantly lower than HMTs with higher hierarchical levels under large impact angle.

**Table 3:** Detailed crashworthiness data of HMTs with different hierarchical levels.

No	*a* (°)	*t* (mm)	*m* (kg)	EA (J)	SEA (kJ/kg)	IPCF (kN)	CFE (%)
HMT-0	0	1.5	0.1660	5529.01	33.31	115.04	47.12
HMT-0	10	1.5	0.1660	3886.34	23.41	18.10	210.48
HMT-0	20	1.5	0.1660	3618.08	21.80	17.22	206.03
HMT-0	30	1.5	0.1660	1943.80	11.71	13.17	144.74
HMT-1	0	1.205712	0.1660	5601.09	33.74	85.77	64.02
HMT-1	10	1.205712	0.1660	5859.86	35.30	15.35	374.33
HMT-1	20	1.205712	0.1660	4160.04	25.06	11.58	352.17
HMT-1	30	1.205712	0.1660	2145.87	12.93	9.81	214.42
HMT-2	0	0.986699	0.1660	6916.91	41.67	67.18	100.95
HMT-2	10	0.986699	0.1660	6400.01	38.55	12.24	512.48
HMT-2	20	0.986699	0.1660	4584.08	27.61	9.02	498.00
HMT-2	30	0.986699	0.1660	3637.53	21.91	4.88	730.55
HMT-3	0	0.9	0.1660	7532.08	45.37	65.40	112.92
HMT-3	10	0.9	0.1660	5702.85	34.35	12.46	448.73
HMT-3	20	0.9	0.1660	4854.09	29.24	7.64	622.92
HMT-3	30	0.9	0.1660	3806.60	22.93	5.76	647.97
HMT-4	0	0.864204	0.1660	8458.52	50.95	63.11	131.40
HMT-4	10	0.864204	0.1660	7301.66	43.99	11.81	606.34
HMT-4	20	0.864204	0.1660	4649.86	28.01	7.22	631.30
HMT-4	30	0.864204	0.1660	3601.94	21.70	5.90	598.87

**Fig. 21:**
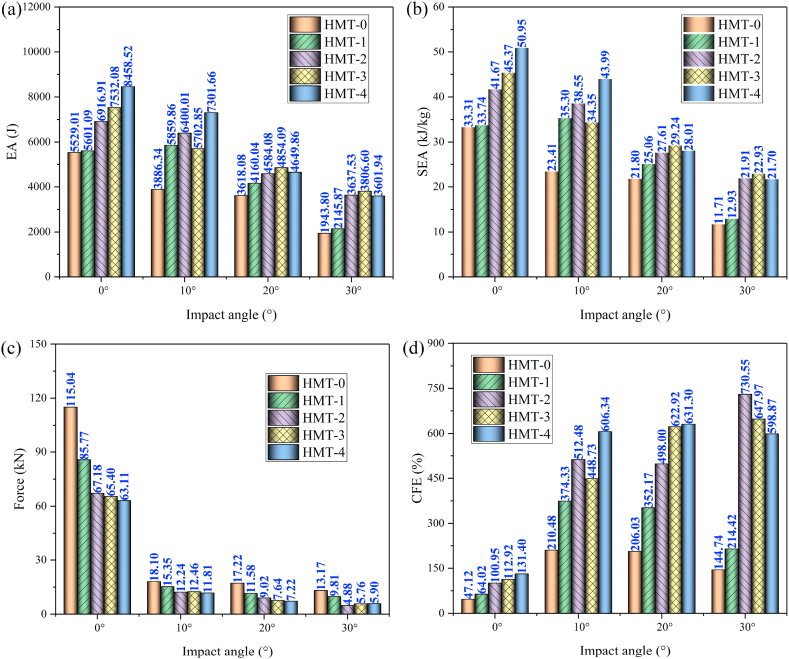
Crashworthiness data of HMTs with different hierarchical levels, (a) SEA, (b) MSEA, (c) PCF and (d) CFE.

Detailed crashworthiness data of HMTs with the same mass is showed in Fig. 21 (a, b, c, d) and Table 3. The tendencies of EA and SEA are the same due to they have the same structural mass. Generally, HMTs with higher hierarchical levels have higher EA and SEA. When the impact angle is small, the EA of structure is better and with the increase of impact angle, the EA and SEA decrease. HMTs with higher hierarchical levels have lower IPCF because of the thinner wall thickness near the impact end. CFE (the ratio of MCF to IPCF) is another key indicator for crashworthiness, which is closely related to the value of MCF and IPCF. Under the large impact angle, the IPCF and MCF of HMTs simultaneously decrease and thus the relationship between CEF and hierarchical levels is irregular. Generally, HMTs with higher hierarchical levels have higher CFE. For example, the CFE of HMT-2 under the impact angle of 30° reaches up to 730.55%. The aforementioned data reflects the superiority of the proposed HMTs in reducing the IPCF and increasing the CFE.

### 4.2 Effect of wall thickness

**Fig. 22:**
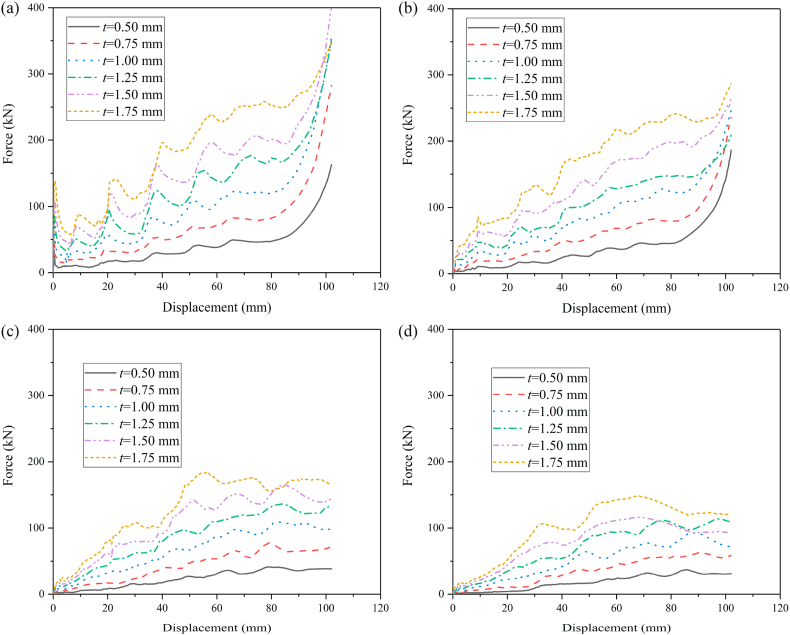
Force-displacement curve of HMTs with various wall thicknesses, (a) 0°, (b) 10°, (c) 20° and (d) 30°.

**Fig. 23:**
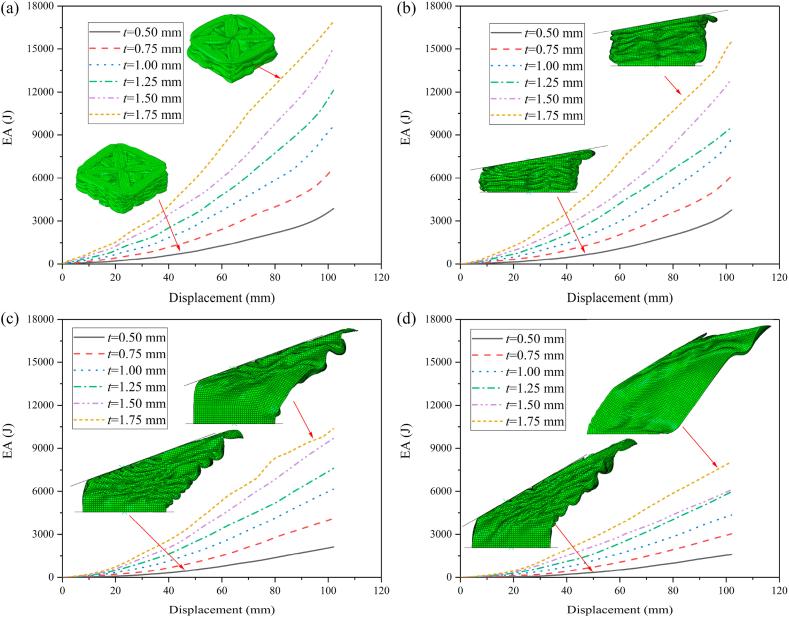
Energy absorption curves of HMTs with various wall thicknesses, (a) 0°, (b) 10°, (c) 20° and (d) 30°.

**Fig. 24:**
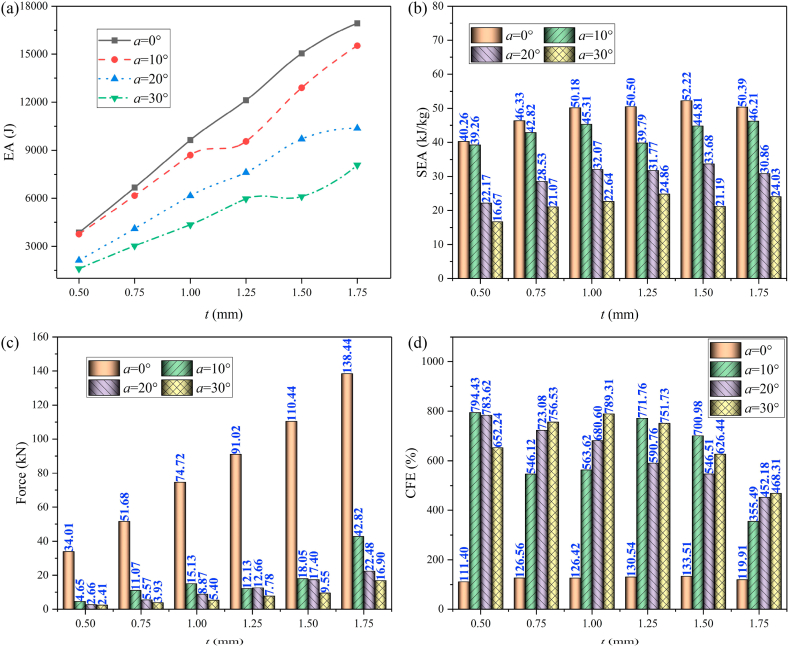
Crashworthiness analysis of HMTs with various wall thicknesses, (a) EA, (b) SEA, (c) IPCF and (d) CFE.

The aforementioned analysis shows that the crashworthiness performances of HMT-4 are better than other structures. Taking the HMT-4 as example, the effect of wall thickness on the crashworthiness performances is investigated and the detailed crashworthiness data is shown in Table 4. The force-displacement and energy absorption curves of structure are shown in Fig. 22 (a, b, c, d) and Fig. 23 (a, b, c, d). The plot shows that thicker wall thickness induces larger impact force and higher energy absorption. However, the force fluctuation is also more severe. The crashworthiness data analysis is shown in Fig. 24 (a, b, c, d). Obviously, with the increase of wall thickness, the energy absorption continuously increases, and the smaller impact angle tends to absorb more energy. The tendency of SEA is completely different from that of EA. The SEA of structure does not increase monotonically because the mass also increases as the wall thickness increases, as shown in Fig. 24b. When the impact angle is equal to 0°, HMT with 1.5 mm is the best in SEA. When the impact angle increases to 10°, 20° and 30°, the best SEA occurs at the wall thickness of 1.75 mm, 1.5 mm and 1.25 mm, respectively. Therefore, unlike the EA, simply increasing the wall thickness cannot always increase the value of SEA. The IPCF increases with the increase of wall thickness. Especially when the impact angle is equal to 0°, it almost increases linearly due to the stronger impact force resistance induced by the increased wall thickness. Fig. 24d shows that all the CFEs exceed 100%. Under larger impact angle, HMT-4 has very high CFE.

**Table 4:** Detailed crashworthiness data of HMTs with various wall thicknesses.

No	*a* (°)	*t* (mm)	*m* (kg)	EA (J)	SEA (kJ/kg)	IPCF (kN)	CFE (%)
HMT-4	0	0.5	0.0960	3864.54	40.26	34.01	111.40
HMT-4	0	0.75	0.1440	6671.88	46.33	51.68	126.56
HMT-4	0	1	0.1920	9635.30	50.18	74.72	126.42
HMT-4	0	1.25	0.2400	12119.57	50.50	91.02	130.54
HMT-4	0	1.5	0.2880	15039.80	52.22	110.44	133.51
HMT-4	0	1.75	0.3360	16932.50	50.39	138.44	119.91
HMT-4	10	0.5	0.0960	3769.16	39.26	4.65	794.43
HMT-4	10	0.75	0.1440	6166.00	42.82	11.07	546.12
HMT-4	10	1	0.1920	8699.69	45.31	15.13	563.62
HMT-4	10	1.25	0.2400	9549.45	39.79	12.13	771.76
HMT-4	10	1.5	0.2880	12906.22	44.81	18.05	700.98
HMT-4	10	1.75	0.3360	15527.54	46.21	42.82	355.49
HMT-4	20	0.5	0.0960	2128.56	22.17	2.66	783.62
HMT-4	20	0.75	0.1440	4108.96	28.53	5.57	723.08
HMT-4	20	1	0.1920	6157.82	32.07	8.87	680.60
HMT-4	20	1.25	0.2400	7625.62	31.77	12.66	590.76
HMT-4	20	1.5	0.2880	9698.61	33.68	17.40	546.51
HMT-4	20	1.75	0.3360	10369.11	30.86	22.48	452.18
HMT-4	30	0.5	0.0960	1600.73	16.67	2.41	652.24
HMT-4	30	0.75	0.1440	3033.97	21.07	3.93	756.53
HMT-4	30	1	0.1920	4347.55	22.64	5.40	789.31
HMT-4	30	1.25	0.2400	5965.34	24.86	7.78	751.73
HMT-4	30	1.5	0.2880	6103.43	21.19	9.55	626.44
HMT-4	30	1.75	0.3360	8074.65	24.03	16.90	468.31

### 4.3 Effect of internode distance

**Fig. 25:**
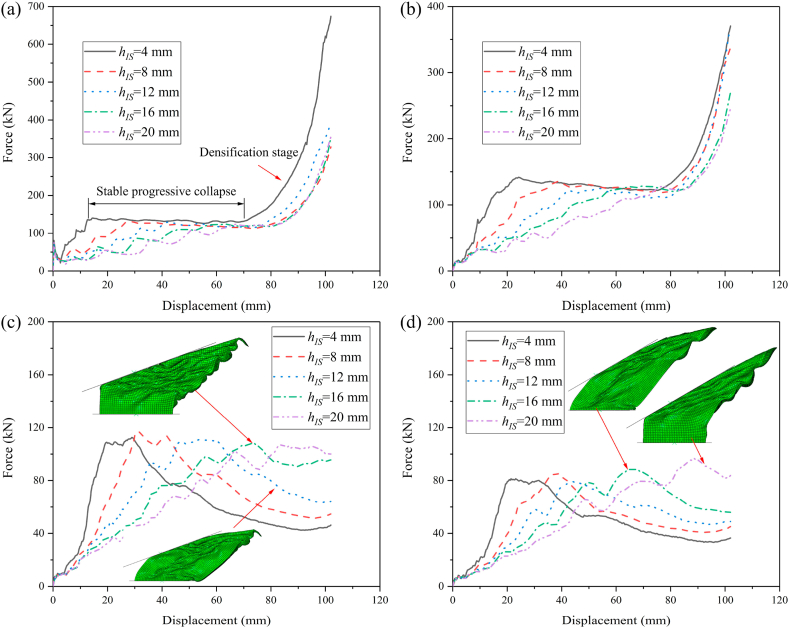
Force-displacement curve of HMT-4 with different internode spaces, (a) 0°, (b) 10°, (c) 20° and (d) 30°.

**Fig. 26:**
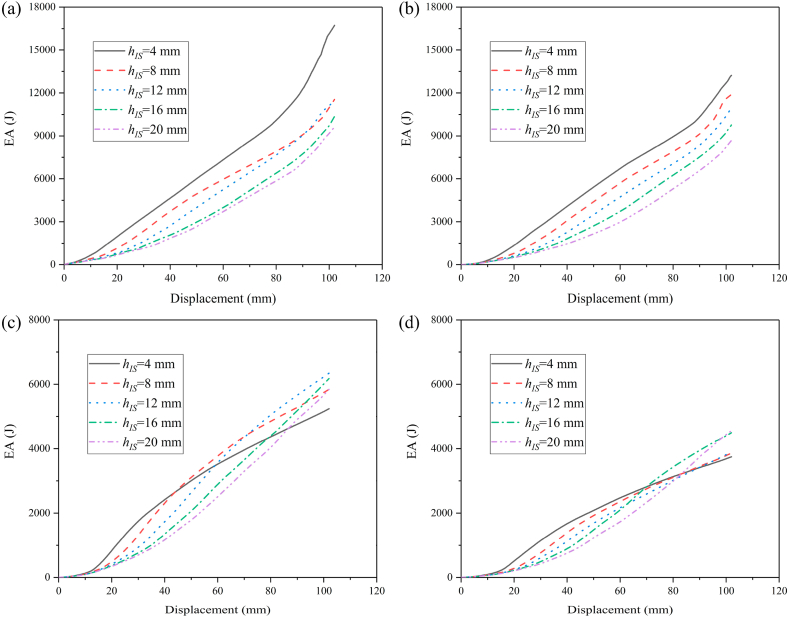
Energy absorption curve of HMT-4 with different internode spaces, (a) 0°, (b) 10°, (c) 20° and (d) 30°.

**Fig. 27:**
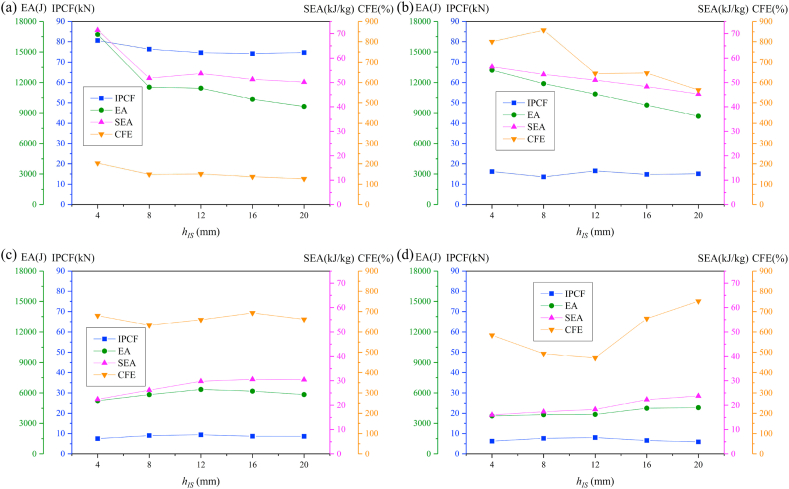
Crashworthiness data of HMT-4 with different internode spaces, (a) 0°, (b) 10°, (c) 20° and (d) 30°.

To investigate the influence of distance of internode space on the crashworthiness performances of HMTs, the internode distances are set as 4 mm, 8 mm, 12 mm, 16 mm and 20 mm, respectively. The force-displacement curve and energy absorption curve are shown in Fig. 25 (a, b, c, d) and Fig. 26 (a, b, c, d). Fig. 25 shows that when the impact angle is equal to 0° and 10°, the impact force increases to a peak value rapidly and then it drops down quickly to a local minimum value. After the valley, the curve gradually increases and enters into a flat distinct plateau region (stable progressive collapse). Finally, the thin-walled structure is completely compacted (corresponding to the densification stage). HMT-4 with smaller internode space reaches into the plateau region and the densification stage more quickly. Whereas, for the large internode space, the impact force will gradually increase after the IPCF, and then enter into the stage of stable progressive collapse. When the impact angle is larger than 10°, HMT-4 with smaller internode space is easier to generate the overall bending deformation mode and the force-displacement curve will present a large single peak, as shown in Fig. 25c and d. Fig. 26 shows that the energy absorption increases as the internode space decreases under the impact angles of 0° and 10°, but the opposite trend is found under the impact angle of 30°. When the impact angle is equal to 20°, the EA increases firstly and then decreases. Detailed crashworthiness data of HMT-4 with different internode spaces is shown in Fig. 27 (a, b, c, d) and Table 5. The plot shows that when the impact angles are equal to 0° and 10°, the SEA increases as the internode space decreases. Whereas, when the impact angles are equal to 20° and 30°, the SEA continuously increases with the increase of internode space. The IPCF almost keeps constant with the change of internode space because the wall thickness and the structure shape near the impact end are almost the same under different internode spaces. For the CFE, it almost remains a low value under the impact angle of 0° due to the relative high IPCF. Under the oblique impact loading, the CFE will significantly increase (up to 800.83%), but the relationship between the CFE and internode space is irregular.

**Table 5:** Detailed crashworthiness data of HMT-4 with different internode spaces.

*No*	*a* (°)	*t* (mm)	*h*_*IS*_	*m* (kg)	EA (J)	SEA (kJ/kg)	IPCF (kN)	CFE (%)
HMT-4	0	1	4	0.2340	16728.09	71.49	80.62	203.42
HMT-4	0	1	8	0.2230	11547.18	51.78	76.39	148.20
HMT-4	0	1	12	0.2130	11442.36	53.72	74.65	150.27
HMT-4	0	1	16	0.2020	10365.04	51.31	74.22	136.91
HMT-4	0	1	20	0.1920	9635.30	50.18	74.72	126.42
HMT-4	10	1	4	0.2340	13220.79	56.50	16.20	800.26
HMT-4	10	1	8	0.2230	11897.55	53.35	13.60	857.75
HMT-4	10	1	12	0.2130	10869.65	51.03	16.53	644.83
HMT-4	10	1	16	0.2020	9767.73	48.36	14.80	646.88
HMT-4	10	1	20	0.1920	8699.69	45.31	15.13	563.62
HMT-4	20	1	4	0.2340	5243.47	22.41	7.56	679.99
HMT-4	20	1	8	0.2230	5840.63	26.19	9.05	633.02
HMT-4	20	1	12	0.2130	6344.68	29.79	9.43	659.88
HMT-4	20	1	16	0.2020	6178.13	30.58	8.73	693.46
HMT-4	20	1	20	0.1920	5845.16	30.44	8.66	661.97
HMT-4	30	1	4	0.2340	3748.84	16.02	6.30	583.74
HMT-4	30	1	8	0.2230	3862.85	17.32	7.68	493.13
HMT-4	30	1	12	0.2130	3895.40	18.29	8.07	473.13
HMT-4	30	1	16	0.2020	4497.29	22.26	6.62	665.88
HMT-4	30	1	20	0.1920	4561.42	23.76	5.95	751.09

## 5 Conclusions

The existing gradient hierarchical tubes only have unidirectional gradient character and they have no advantage in reducing the initial peak force. To decrease the initial peak force of hierarchical tubes and maintain their high energy absorption characters, this paper proposes the bidirectional gradient hierarchical thin-walled tubes by mimicking the structure of bamboo stem. The proposed hierarchical multicellular tubes (HMTs) have gradient characters along axial and radial directions. Their crashworthiness performances under the oblique loads are systematically carried out. The following conclusions can be made.

(1) Compared with the square tube with the same mass, HMTs have higher energy absorption capability under different impact angles. The maximum increases of specific energy absorption (SEA) and crush force efficiency (CFE) reach up to 67.02% and 806%, respectively. Whereas, the maximum decrease of IPCF reaches up to 79.92%.
(2) Unlike the conventional thin-walled tubes, the impact force of HMTs gradually increases as the impact distance increases under the impact angle of 0°. Under the oblique impact loading, the larger fold number and shorter fold wavelength are found for HMT-3 and HMT-4 compared with square tube, resulting in superior energy absorption performances.
(3) HMTs with higher hierarchical levels have better energy absorption capability and lower initial peak crush force. The relationship between hierarchical level and CFE is irregular, but generally, the CFE of HMTs with higher hierarchical levels is higher than that with lower hierarchical levels.
(4) As the wall thickness increases, the energy absorption and initial peak crush force (IPCF) of HMTs increase. However, the relationship between the SEA and the wall thickness is irregular. It must be pointed out that HMTs with different wall thicknesses have a very high CFE under large impact angle.
(5) HMTs with smaller internode space tend to enter into the plateau region and the densification stage more quickly. Moreover, under the large impact angle, they tend to produce overall bending deformation mode. When the impact angle is small, the energy absorption decreases as the internode space increases and the opposite tendency is found under the larger impact angle. The initial peak crush force almost keeps constant with the increase of internode space.

## Authors’ contributions

Xiaolin Deng, Fuyun Liu and Jiale Huang conceived and designed the experiments. Xiaolin Deng, Qi Lu and Qiuyun Wei performed the experiments. Xiaolin Deng, Mei Liang and Jiale Huang analyzed and interpreted the data. Xiaolin Deng contributed reagents, materials, analysis tools or data. Xiaolin Deng, Qi Lu, Mei Liang, Qiuyun Wei and Jiale Huang wrote the paper.

## Declaration of competing interest

The authors declare that they have no known competing financial interests or personal relationships that could have appeared to influence the work reported in this paper.

## Data availability statement

All the data needed to evaluate the conclusions in the paper are present in the paper.
